# Neural network-based identification for scallops (*Pecten maximus*) in natural marine habitats

**DOI:** 10.1371/journal.pone.0327824

**Published:** 2025-07-28

**Authors:** Leander Harlow, Katja Ovchinnikova, Mark James

**Affiliations:** 1 UHI Shetland, Port Arthur, Scalloway, Shetland, United Kingdom; 2 Department for Biomedical Research, University of Bern, Bern, Switzerland; 3 University of St Andrews, School of Biology, St Andrews, United Kingdom; Bigelow Laboratory for Ocean Sciences, UNITED STATES OF AMERICA

## Abstract

The Great Atlantic scallop, or King scallop (*Pecten maximus*), ranks third in value after mackerel and Nephrops in UK fisheries. Its landings have surged over recent decades, making it the UK’s fastest-growing fishery. Scallop stock assessments, crucial for sustainable fisheries management, traditionally rely on fisheries surveys, including underwater imaging and dredge sampling. Data on areas that contain scallops but not fishable using dredges is lacking. Dredge sampling is also potentially destructive. Remote data collection using drop down cameras and towed video are used, but there are few tools available to analyse these data automatically. *P. maximus* are usually recessed in fine sand and gravel habitats making image identification challenging. This study explores the potential of Artificial Intelligence (AI), specifically the NetHarn model from the VIAME toolkit, to identify and count scallops from underwater video transects. The research utilises diverse video footage from NatureScot, captured with custom camera systems (DDV and miniDDV), providing varied habitat, image quality, and camera specifications. Previous AI studies of this species artificially placed scallops on the seabed and are not representative of natural presentation. This research applies the same AI model to survey images featuring scallops in their natural habitat. Results showed moderate performance of the NetHarn model, achieving an F1 score of 0.44 and a mean Average Precision (mAP) of 0.41 when classifying scallops into three categories: king, queen, and dead. Model performance varied across geographic locations, camera platforms, and habitat types, with challenges including blurred images and mislabelling. The study emphasises the need for improved data acquisition, standardised camera systems, and larger annotated datasets to enhance AI model performance. Despite moderate results, this research highlights AI’s potential for automating estimation of scallop stock abundance and marine habitat monitoring. Future efforts should focus on addressing image quality issues, increasing sample sizes, and optimising data collection for enhanced marine conservation and fisheries management.

## 1 Introduction

The Great Atlantic scallop or king scallop (*Pecten maximus)(scallops)* first sale value ranked third, following that of mackerel (*Scomber scombrus*) and Nephrops (*Nephrops norvegicus*) and is the fastest growing fishery in the UK. Landings for king scallops increased from 14 to 131 thousand tonnes between 1994 and 2021 [1,2]. The first sale value in 2017 equated to £68.7 million, generated by landings in excess of 26,800 tonnes [3]. While landings mainly comprise king scallops, queen scallops (*Aequipecten opercularis)(queen)* are also significant as the UK landings were the highest in Europe, with an average of around 6,000 tonnes each year between 2004 and 2009 [4]. Twelve thousand tonnes of queen scallops were landed in 2010, with a value of £4.6 million [5]. The queen fishery is in decline where 17 tonnes were landed by UK vessels in the EU in 2021 [1].

Scallop stock assessments are carried out as part of statutory monitoring programmes fulfilling a range of policy requirements including for example the European Common Fisheries Policy, and the Marine Strategy Framework Directive. Assessments provide estimates of abundance and population structure with a view to informing the management of the fishery and set reference points with respect to the amount that can be caught, helping to ensure that the fishery remains sustainable over time. Typically, these assessments will involve both fisheries dependent and fisheries independent surveys, the latter often involving the use of underwater imaging of the seabed using towed cameras along defined transects. The resulting images are manually reviewed and any scallops identified and abundance per unit area then estimated. Fisheries independent surveys also involve towed dredge sampling which mimics commercial dredging practice but predicated on the use of standardised dredges deployed in a consistent manner. By definition, this process causes similar seabed disturbance as commercial scallop dredge fishing albeit on a much smaller scale. Stock assessment surveys are usually limited to those areas that are either commercially fished for scallops or in un-fished areas that are suitable for dredge surveys.

Rocky substrates that are unsuitable for dredge surveys, but still likely to contain scallops, are generally not included. The extent to which this may confound stock assessment is unknown. Current assessments largely only cover areas targeted by fishing, and substantial areas of the UK are data-poor [2].

The king scallop is a large bivalve mollusc (up to 175 mm shell length, or 153 mm shell height) that is resident on the continental shelf of Northwest Europe. It is common at depths of 5–200 m, on substrates ranging from muddy sand to coarse gravel. The species ranges from northern Norway to Morocco, the Canaries and the Azores. Scallops are common around the British Isles [6].

The queen scallop (up to ~90 mm shell width) and ranges from south of Norway to the Mediterranean and the Canary Isles and are common around the British Isles. It is commonly found sub-tidally to depths of 100 m and on sand or gravel, often in high densities [7].

King scallops usually recess into the substrate, being covered by a thin layer of sand or gravel, with only the margin of the shell and occasionally the fringing tentacles of the mantle being visible. Where the shell is not covered, the ribbed surface of the shell can be easily identified, but visually differentiating between dead and living specimens can be difficult. Queen scallops live on the surface of the substrate, can be highly mobile and are easily identified by both their shape and colouration. Visually differentiating between living and dead specimens is relatively simple as characteristic and often brightly coloured epifaunal sponges are lost post-mortem (James, pers com.). Differentiating between king and queen scallops is also typically straightforward as they are usually different in size, shape, colour and position in, or on the substrate. However, the ribbed nature of the shell, similarities in shape and the inability to determine size in the absence of range in 2D images can lead to misidentification when these species are only partially visible (James, pers coms).

The use of Artificial Intelligence (AI) is already widespread in estimating the population density of specific target species, a process essential for ecological research and fisheries management, as well as for broader environmental conservation efforts that focus on habitat protection and species preservation [8–16]. Li et al [17] successfully used the YOLOv5s model to identify cultivated scallops in a laboratory environment with high detection results. Similarly, Natsuike et al [18], used another deep learning model to recognise scallops in lantern nets used for aquaculture.

Scallop detection was investigated by [11] used multiple features and a series of cascaded Adaboost classifiers, to develop one of the most prominent scallop detection algorithms. A more recent attempt was presented by Rasmussen et al [19] who tested variations of the YOLOv2 CNN trained for scallop detection. They achieved high accuracy while being able to run in real-time from live recordings from an Autonomous Underwater Vehicle (AUV).

Most recently, efforts were made to standardise the use of AI to support scallop stock assessments [20], by applying the NetHarn_1_class model. Seabed footage was captured by divers using GoPro cameras and by a remotely operated vehicle (ROV) camera system and analysed in the open-source desktop application Video and Image Analysis for Marine Environments (VIAME) [21,22]. This study artificially placed scallops on the seabed to capture images and whilst demonstrating the potential utility of using AI, the scallops were not fully recessed and therefore not representative of a natural situation, however the performance was promising achieving Precision 0.97, Recall 0.95, F1 Score of 0.96, mAP 0.91, with a confidence threshold of 0.5. However, the substrates in the original study do not fully reflect the heterogeneity often characteristic of king and queen scallop habitats.

The aim of our research was to assess the potential to apply the VIAME model to the challenge of identifying and counting naturally occurring king and queen scallops from towed underwater video transects and inform future data collection processes, with a view to automating and improving the efficacy and efficiency of scallop stock assessment methods.

## 2 Materials and methods

The video footage used in this research was provided by NatureScot, an executive non-departmental public body of the Scottish Government and consisted of video line transects designed to capture data to be used for benthic habitat assessments. Necessary details from the original report [23] are provided below. Sites were established based on Marine Protected Areas (MPA’s), accessibility and previous sampling knowledge. Multi-beam sonar seabed mapping determined exact locations at each survey site. Survey’s span across seven sites, including Shetland, Orkney, Jura, Inner Sound and Loch Alsh, South Arran, Small Isles and Wester Ross.

Video footage was captured using one of two systems. A bespoke drop-down video tow developed by C-Technics Ltd (DDV) and a smaller less sophisticated system (miniDDV). For the DDV the submersible tow rig consisted of the primary camera, lights, and scaling lasers. Combined with the surface control unit, operators have the capacity to control the video recording, lighting, and flash settings. The DDV tow was deployed from a chartered fishing vessel along transects specified by NatureScot for the purposes of habitat assessment. Once the DDV was deployed the vessel was allowed to drift in the prevailing wind or currents for ~ 5 minutes and its track coordinates were recorded. Lighting was monitored and kept at, or just below, the maximum ambient levels. With a resolution of 1920 x 1080 pixels, lens focal length of 6.7 mm, and +/- 72-degree tilt, the field of view equates to ~3.58 m by ~2.39 m when the camera is ~ 1 m above the seabed (maintained using an integrated pressure sensor). With the camera oriented perpendicular to the seabed this results in a field of view of ~8.55 m2.

The miniDDV is a smaller system utilising a GoPro Hero 4 camera in a deep-water housing unit and programmed to take HD footage at 60 frames per second (fps). In addition, two white LED light clusters are attached for seafloor illumination along with Outland Technology UWL- 810 red lasers set 100 mm apart for object scaling. The miniDDV was connected to the surface via a 100 m umbilical. The mini-DDV was orientated at a forward angle (unspecified) rather than directly facing the seabed. With a resolution of 2704 x 1520 pixels, lens focal length of 2.92 mm, the field of view underwater equating to ~2.25 m by ~1.38 m when the GoPro camera is positioned ~ 1 m off the seabed. The resulting field of view is ~ 3.49 m2. Although two camera systems with different resolutions were utilised, the images are temporarily downsampled to a consistent resolution during model training for uniformity. The default settings in the VIAME web interface resizes frames to 640 x 640 while an image is smaller than 1.6 mp.

In an attempt to achieve an unbiased representation of all surveyed sites, 47 videos were randomly selected across all sites, ensuring each site was sampled at least once and each habitat was represented, totalling approximately 6 hours and 30 minutes of footage. Habitats present in the footage include circalittoral mixed sediment (SS.SMx.CMx), circalittoral sandy mud (SS.SMu.CSaMu) and sublittoral coarse sediment (SS.SCS).

Images (jpeg) were annotated and subsequently analysed using the VIAME DIVE, a free and open-source annotation and analysis platform (see: https://kitware.github.io/dive/).

Uploaded videos were converted to still images at 1 frame per second (fps), producing 19,857 images in total. Five, randomly chosen, videos were analysed for the best frame extraction rate based on the total number of scallops in the video compared with the number of annotations produced at different frame rates (1, 5 and 10). This ensured no significant overlap of individual scallops in each frame whilst also reducing the chance of missing individuals. On the DIVE interface, still images were grouped into folders by their video geographic origin and randomly split into training (35 videos) and testing (12 videos) subfolders. This stratified random sampling ensured the split was even across survey sites and that each site appeared at least once in both training and testing. In addition, several ‘empty’ frames, or negative samples of various marine habitats containing no scallops, dead or alive, were included in the model training to help reduce incorrect predictions

### 2.1 Annotation

**Fig 1:**
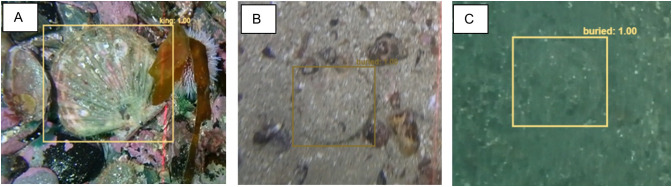
Image example of scallop on the surface, clearly visible in A, partially visible in B and fully recessed in C.

For annotation, we used the VIAME DIVE interface. Rectangular bounding boxes were drawn around each object of interest, which was then assigned to a class; “king”, “buried”, “queen” or “dead”. The classification “buried” refers to recessed king scallops. All dead shells were classified together due to the difficulty of accurate identification between dead king and queen, especially where the shells are in two parts. Fig 1 illustrates the visual difference between a king scallop on the surface of the sediment and when recessed.

To assess the potential for annotator bias, two annotators with experience of identifying scallops underwater annotated the same video independently for comparison.

### 2.2 Model training and testing

The NetHarn model used in this study (VIAME, detector netharn cfrnn) and by Ovchinnikova et al [20] was a Cascade Faster R-CNN [24,25], which addresses two object detection challenges; recognition and correct localisation of the target object [26]. The model had been previously trained specifically for scallops [20] and achieved the best results (0.96 F1 score and 0.91 mAP) and significantly outperformed the model provided by VIAME trained for Atlantic Sea Scallop (*Placopecten magellanicus)* (0.91 vs 0.69 mAP). In this study, three new detection models were trained using ImageNet backbone, with three levels of class aggregation: 1. queen, (surface) king, buried (king), dead; 2. merged label for king scallops (queen, king, dead); 3. merged label for living scallops (alive, dead). The rationale for this aggregation was to assess how well the different classes could be resolved. The trained models were then tested on the test dataset. As a standard process VIAME DIVE conducts augmentation, including rotations, hue shifting, intensity shifting, adding random masks and gaussian smoothing/sharpening to artificially expand the dataset.

To assess how the model’s performance improved as more training data was used, we divided the entire training dataset into six equal subsets. We then trained six models, each with an increasing amount of data. The first model was trained using only the first subset, the second model was trained using the first and second subsets combined, and so on. By the sixth model, all six subsets (the entire training dataset) were used. This approach allowed us to see how performance changed as we gradually increased the size of the training data.

### 2.3 Evaluation metrics

The following evaluation metrics were used in this study and are documented in full in Ovchinnikova et al [20]. Precision (*P*) is the percentage of predicted scallops that are correctly identified and match the ground truth, defined by:


$$P=\;\frac{True\;positives\;}{\left(True\;positives)+(False\;positives\right)}$$

Recall (*R*) or sensitivity is explained as a percentage of the ground truth scallops correctly predicted:


$$R=\;\frac{True\;positives}{\left(True\;positives)+(False\;nagatives\right)}$$

The F1 score is the harmonic mean of the precision and recall defined as follows: $F1=2*\;\frac{P*R}{P+R}$

The highest possible value of an F1 score is 1, indicating perfect precision and recall, and the lowest possible value is 0, if either the precision or the recall is zero. If the predictor produces a confidence score, then a threshold on this score can be used to filter out unreliable predictions. Correct prediction is defined using the Intersection over Union (IoU) score. In our experiments, we considered IoU>= 0.5 to be a match. Following the standards for PASCAL VOC (2020; http://host.robots.ox.ac.uk/pascal/VOC/) object detection challenges [27], the mean average precision (mAP) was calculated, which computes the average precision value for recall levels ranging from 0 to 1 with step 0.1.

Frames containing false positives were manually inspected to conduct error analysis. This included in-water column detections where there was “no seabed”, “partial” scallops that were not fully in frame or covered by other benthic fauna, detections that were correct but missed in the annotation stage (thus errors of the annotator and not of the model) and incorrect detections caused by blurred images or similarly shaped objects such as stones or sea urchins. In addition, frames where scallops were present but not detected by the model, were classified into four main error classes: blurred, dark, partial, or recessed. In some instances, there were multiple reasons for an error, in which case two classes were listed leading with the most pronounced error.

## 3 Results

### 3.1 Annotated dataset

In total, 47 videos were annotated, with 30 videos (71%) selected for training and 12 (29%) selected for testing the models (Table 1).

**Table 1:** Number of annotated scallops labelled with each class label.

	Queen	King		Dead
		On surface	Buried	
Training set	5382 (65%)	498 (6%)	810 (9%)	1608 (19%)
Test set	311 (50%)	78 (12%)	150 (24%)	90 (14%)
Total	5679 (68%)	446 (5%)	590 (7%)	1653 (20%)

One video (EMFF_LochAlsh2018_Station_70_2018_07_20) was annotated by two independent annotators. In total, 2147 and 2304 scallops (king and queen) were found by annotator 1 and annotator 2, respectively. Out of all annotations, 73% matched between both annotators (IoU>= 0.5). For matching annotations, 98% of class labels were matching. Where annotator 2 had more experience identifying scallops in their natural habitat, annotator 1 did not detect or label all the scallops present. This result shows that the task of detecting scallops with reasonable consistency in the selected videos was feasible. Queen scallops were much more abundant in the data set representing ~85% of the annotated live scallops (king and queen).

### 3.2 Model evaluation

The detection model trained with just two labels (alive and dead) performed best in terms of mean Average Precision (mAP = 0.42), recall (0.39) and accuracy (F1 = 0.44) scores (Table 2). Introducing more classes slightly lowers the model’s performance (mAP = 0.41).

**Table 2:** Percentages of scallops in each class detected as alive, dead or not detected by the detection model using 2 classes (alive/dead) for training.

Class	Number of observed scallops	% detected alive	% detected dead	% not detected
queen	78	0.51	0.04	0.45
surface king	150	0.44	0.02	0.54
buried king	311	0.33	0.01	0.66
dead	90	0.01	0.30	0.69

The model performance results, evaluated at a confidence threshold of 0.9, indicate a clear variation in detection accuracy between different scallop conditions (Table A1). Specifically, dead and buried scallops exhibited the lowest detection rates, suggesting challenges in identifying these categories with high confidence. In contrast, queen scallops were detected with the highest accuracy, reflecting the model’s robustness in identifying this class.

Precision and recall graphs are valuable for assessing classification model performance, especially with imbalanced datasets where accuracy may not provide a complete picture. A higher area under the curve (AUC) demonstrates better model performance. Where precision and recall have an inverse relationship, there is a trade-off between increased recall and lower precision as more false positives are seen in the models’ predictions.

**Fig 2:**
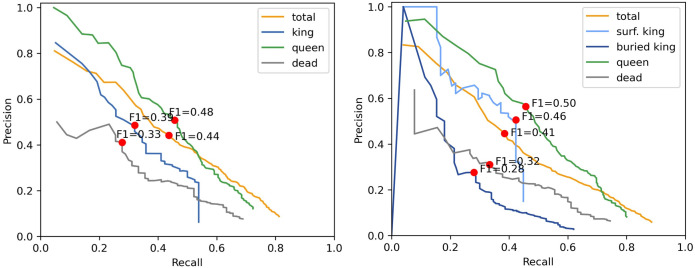
Precision-recall curves. Models trained with labels queen/king/dead (left) and queen/surface king/buried, king/dead (right). Red dots show precision-recall trade-off with the best F1 score for each class.

The three-class model performs better overall, with “queen” achieving the highest AUC, a result also reflected in the more complex model. The rapid drop in precision and recall for “king” (left plot) and “surface king” (right plot) indicates that lowering the threshold to include more potential positives suddenly admits a large number of false positives. Conversely, at very high thresholds, the more complex model fails to predict any instances of “buried king,” causing the precision–recall curve to start at the (0, 0) origin (Fig 2, right). As expected, buried kings have a lower chance of detections compared to surface kings.

Separation of surface and buried king scallops did not confer any advantage for scallop detection (Fig 2).

**Fig 3:**
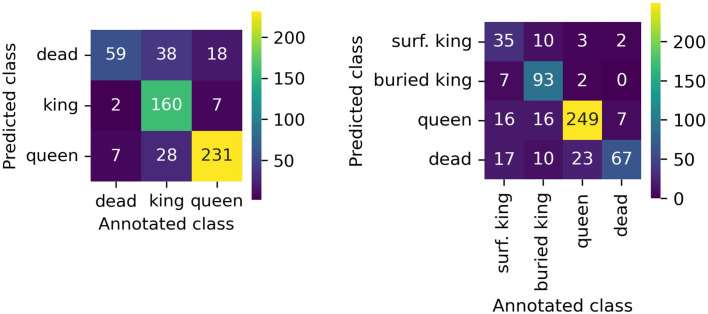
Confusion matrices for the models trained with labels queen/king/dead (left) and surface king/buried king/queen/dead (right). The values show how many scallops annotated with each class were detected as the same or different class.

Model predictions generally align with the true class, along the diagonal, for both models, indicating they can distinguish each class with reasonable accuracy. Queen scallops were the easiest to detect and label correctly in both experiments. This results from the fact that most of the annotated scallops were queens, reflecting their relative abundance compared to king scallops in the videos annotated (Table 1). In both experiments, king scallops were the most difficult to label correctly (Fig 3). Off-diagonal numbers show there is some confusion, for example, dead occasionally gets predicted as king and queen is sometimes misclassified as surf. king or buried king. The best total F1 is 0.44, precision 0.44 and recall 0.44 with a confidence threshold 0.81 (S1 Appendix). This suggests that while the model captures most key distinctions, more training data or targeted refinements may be needed to address these recurring misclassifications.

**Fig 4:**
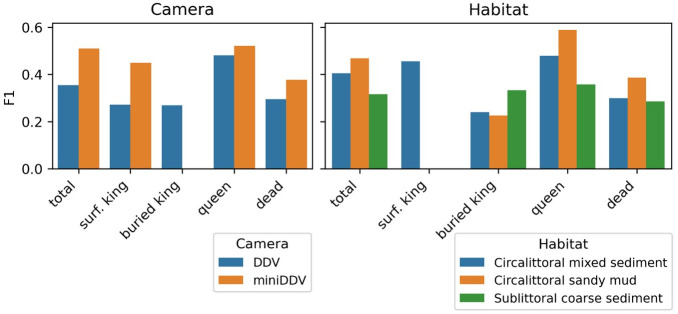
F1 scores for all classes (surface king, buried king, queen, dead). Confidence threshold of 0.89 is selected, based on the type of camera used and habitat type.

Buried kings were more likely to be identified by the model in footage taken with the larger DDV, compared to the miniDDV (Fig 4). In the DDV footage 122 predictions were made from a total of 134 annotations, whereas the model was unable to accurately predict any of the sixteen individuals annotated in the miniDDV footage. There was no difference in the model’s ability to predict surface kings for the DDV, unlike the miniDDV where the F1 score is much higher than for buried scallops. Similarly, in the habitat comparison, some classes (queen) show relatively high F1 scores across habitats, while others (buried king) appear more sensitive to changes in substrate (Fig 4). These differences highlight that both hardware (camera choice) and environmental context (habitat type) can influence the model’s ability to identify scallops accurately.

**Fig 5:**
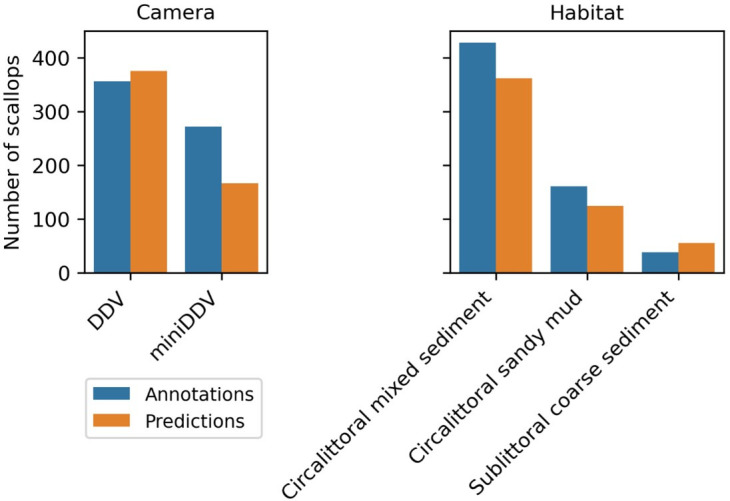
Number of scallops annotated compared to scallops detected using the model with the best F1 score as a function of location (transect), type of DDV used and habitat type.

The model predicted fewer scallops compared to the annotated count in footage captured by the miniDDV camera (Fig 5). However, only two videos were analysed from the miniDDV, both filmed in habitats also surveyed with the DDV system. Therefore, the observed discrepancy may be due to the limited sample size rather than inherent differences between camera systems

The model performed the best where footage contained circalittoral sandy mud habitats (Fig 5), where fewer rocks, pebbles and shells are typically found compared to circalittoral mixed sediment areas. Similarly, the model over predicted abundance for sublittoral coarse sediment areas, perhaps due to a greater presence of more coarse materials such as gravel, pebbles, or shell debris which the model may confuse for a scallop. Overall, the presence or absence of turbidity, the degree of scallop burial, and the abundance of shell fragments or rocks all influence how many scallops the model predicts.

A notable gap between bars would suggest that the model either under- or over-predicts certain classes for a given camera or habitat (Fig 5). These insights, in conjunction with the confusion matrices, can guide future improvements—for instance, collecting more training examples in underrepresented conditions, adjusting sampling strategies, or refining class definitions where confusion is consistently observed.

Incremental increases in the training data resulted in a corresponding improvement in the model’s performance, demonstrated by a steady rise in mean average precision (mAP). This suggests that the model performance can potentially be further improved by adding more annotations, especially of king scallops.

### 3.3 Error analysis

**Fig 6:**
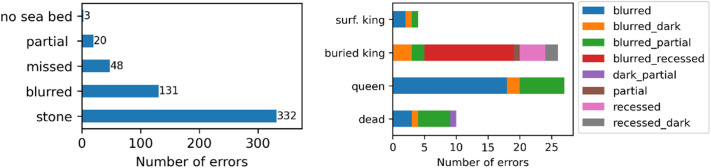
Number of analysed false positives by error type (left) and undetected scallops by species and error type (right).

**Fig 7:**
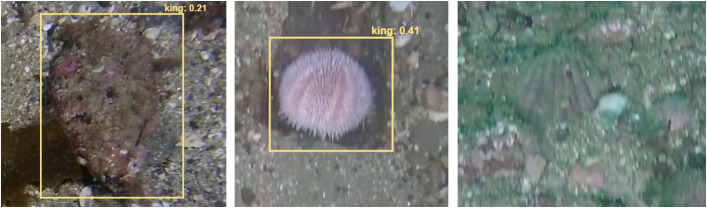
Image examples of detection errors. False positives classifying a stone and an urchin (left, middle) and a false negative (left) where a partial recessed scallop was missed in detection due to blurred footage.

We analysed false positives in 327 frames of 12 videos, resulting in 538 false positives. Fig 6 (left) shows the number of false positive detections categorised by the type of error. Fig 6 (right) shows scallops not detected by the algorithm further disaggregated as a function of annotated classification; king scallops on the surface (surf. king), recessed (buried king), queen and dead. Most false positives occurred where other objects, such as rocks or sea urchins, with a similar shape were predicted as scallops (Fig 7). The second highest error category was due to low quality images, namely blurred or poorly illuminated images (Fig 7). This is reflected in the number of detection errors for recessed king scallops and queen scallops. Unsurprisingly, for king scallops, the most frequent error occurred where the scallop was recessed in conjunction with poor image quality.

## 4 Discussion

The performance scores for the best NetHarn model, including buried and surface kings, detailed in this research are moderate with a F1 score of 0.44 and mAP 0.41. The model detected 44% of the scallops with the optimal confidence threshold. The model with fewer classes (dead/alive) outperformed (mAP = 0.42, F1 = 0.44) those with more classes (kind/queen/dead and surface king/buried king/queen/dead), perhaps owing to the cryptic nature of buried scallops. In particular, queen scallops were detected with the highest accuracy, while dead and buried scallops showed significantly lower detection rates, reflecting difficulties in confidently distinguishing these categories. Moreover, the analysis revealed that detection performance varied with habitat type and camera system, the model performed best in circalittoral sandy mud habitats, likely due to fewer confounding objects such as rocks and shells, whereas sublittoral coarse sediment areas led to over-predictions, possibly because gravel, pebbles, and shell debris were misinterpreted as scallops. Finally, incremental increases in training data corresponded with improvements in mAP, suggesting that further annotations, particularly for challenging classes like buried scallops, could enhance the models accuracy.

The results suggest that using conventional seabed video transects used for habitat identification purposes in heterogeneous environments to detect cryptic species, in this case recessed king scallops (*P. maximus*), remains a significant challenge. Ovchinnikova et al [20] document the limitations of underwater imaging that result in poor image quality, namely sediment, light, and unfocused images as a result of camera movement. Whilst artificial light can be used together with faster shutter speeds to address motion blur, sediment in the water can be reduced but not eliminated by decreasing the camera to subject distance. However, the results clearly demonstrated the ability of the algorithm to detect both surface and fully recessed scallops where image quality was not compromised. Where images were in sequence, the annotator had an advantage over the algorithm in detecting inconspicuous scallops due to poor image quality and where they were recessed.

Unsurprisingly blurred images and blurred images of recessed king scallops were the largest sources of error. The videos used in this study were not specifically recorded for the purpose of scallop stock assessment on grounds that are commercially fished for scallops. It is likely that the challenges presented by the highly diverse benthic structure and community composition found in many of the videos reviewed as part of this research, represent a more significant challenge for the algorithm than a more homogeneous coarse sandy substrate that may better represent a dredged scallop bed. In many respects the transect images used in this study could be an analogue for the ground exploited by scallop divers. Such grounds are also unlikely to form part of conventional stock assessments as they would be unsuitable for dredging. They do, however, represent locations where scallop populations have not been assessed as part of the overall stock and their significance as sources of scallop production are unknown.

The error analysis of the model used in this research highlights the need to consider some key variables. Two different camera systems were used which clearly had different imaging attributes both in terms of control over proximity to the subject (seabed) and the angle at which images were recorded. Differences in habitat type influenced the abundance of the target species of interest, but the results suggest that this was not responsible for major differences in actual and predicted counts of scallops. Different geographic locations showed similar trends if not absolute agreement between actual and predicted counts. The misidentification of stones and stone-like objects as scallops, assuming that the images were clear, would support the need for increasing the size of the training set for buried scallops in particular. Blurred images of recessed scallops are also an important source of error. Blurring may result from the image being out of focus and or passing through the frame at a speed that exceeds the frame rate. These factors would require that the camera platform be more carefully controlled in terms of both speed and distance to subject.

False positives and false negatives in automated detection models have direct implications for abundance estimates used in stock assessments. Overestimating scallop presence due to false positives could lead to inflated population estimates, potentially increasing the Total Allowable Catch (TAC) beyond sustainable levels. Conversely, false negatives, or missed detections, could result in underestimates, prompting unnecessarily conservative management. The magnitude and direction of these errors matter depending on the type of model used in assessments, which may include density-based approaches, distance sampling, or occupancy models. While this study does not integrate model outputs directly into stock assessments, the prevalence and nature of prediction errors should be considered when determining the reliability of AI-assisted survey methods. There is currently no internationally agreed threshold for “acceptable” detection error in marine stock surveys; tolerance for error is typically determined by the management authority and balanced against cost and risk. Generally, greater uncertainty leads to more precautionary TACs.

State-of-the-art algorithms are becoming mature enough to provide automated analysis when applied to relatively controlled situations such as aquaculture [18,28–30]. Gladju et. al [31] reviews data mining applications and machining learning frameworks in fisheries and aquaculture. However, the review focuses on applications in aquaculture and fish processing, both of which are, by definition, relatively controlled environments and processes. The application of AI in natural and highly variable environments such as in capture fisheries for example, is more challenging. Pedersen et al [32] provide a generic study in natural conditions in brackish water classifying a broad range of taxa but also attempt to address the challenges of poor image quality resulting from blurred images, which can be a feature of brackish conditions, sediment and backscatter.

Increasing access to integrated AI platforms such as VIAME DIVE (https://kitware.github.io/dive/), Roboflow (https://roboflow.com/), V7Labs (https://www.v7labs.com) or FathomNet (FathomNet) for example opens up the opportunity for non-specialists to embrace the promise of AI and invest considerable time and resources in annotating many thousands of images and subsequently training a range of AI models, some with relevant pretrained backbones. However, to fully realise the promise of AI in challenging marine environments, investment in optimising the acquisition and annotation of data suitable for AI analysis needs to be carefully considered and suitably resourced from the outset. The fact that we can potentially access increasingly large volumes of data does not, of itself, necessarily mean that we can use it to train reliable AI models.

A major constraint in using opportunistically derived datasets for training machine learning models is the lack of control over the manner in which the images are collected. It is interesting to note that the video transects used in this study were originally recorded for the purposes of habitat assessment which involves experienced human observers manually reviewing the footage to define benthic habitat types including particular marine features which may, for example, include protected marine features such as maerl beds [33]. There is growing interest in the use of AI and machine learning to automate monitoring of the marine environment for both conservation and management [34]. Automated processing of image-based data from coral reefs using machine learning technologies for example resulted in a 99% cost reduction over traditional methods, at 200 times the speed [35]. The promise of automated monitoring, particularly where low-cost open-source processes and systems could be applied has considerable appeal, opening the potential for data acquisition and interpretation and meaningful spatial and temporal scales.

The rate at which ecosystems are likely to change as a function of climate change, demands that relevant data will need to be collected and interpreted at unprecedented scales to support more dynamic decision making, often by non-specialists. However, image and video-based AI remains a considerable challenge in the marine and aquatic environment more generally as it is highly reliant upon the acquisition of imagery that is sufficiently well resolved to effect reliable object detection and classification. Ovchinnikova et al [20] achieved performance of 0.96 F1 score and 0.91 mAP for the identification of *P. maximus*, but this was for scallops that had been artificially placed on the substrate and may not have recessed to the point of burial. The habitat and natural occurrence and presentation of *P. maximus* used in the current analysis indicates the importance of using such data for training purposes but also suggests that a more structured experimental design with respect to data collection is required. There is a general acceptance that a “large” amount of data is required to train a reliable AI network, but the cost and time involved in securing sufficient data of appropriate quality needs to be considered from the outset.

In other areas of research, sample size requirements are usually calculated *a priori*. The performance metrics presented herein, represent a *post hoc* analysis, which could, in principle, be used to inform how many more samples, in this case videos of a particular habitat type and class (scallop) annotations that would be needed to ensure a given level of performance. Estimating the time and resource costs of achieving this then becomes a tangible calculation. An accepted wisdom is that the data available for AI network development is split into training, validation and test sets with training data sets being ~70% −80% of the available data with the remainder reserved for validation and testing [36].

With each successive augmentation of the training dataset, mAP values showed incremental gains, indicating that the model benefitted from exposure to more diverse examples, thereby enhancing its ability to accurately detect scallops across varying conditions. This trend suggests that the model’s performance is data-dependent, and further improvements could be achieved with additional, high-quality training data. This relationship could be used to provide an estimate of the likely sample size required to achieve an acceptable level of model performance. The practicalities and resource costs of achieving this can then be carefully considered. In this case, acquiring a representative training set to reliably detect recessed *P. maximus* across its known habitat range in variable sampling conditions could potentially involve considerable dedicated vessel, equipment and personnel time.

## Supporting information

S1 Appendix S1 Table. Evaluation of 3 scallop detection models trained with 3 different sets of class labels. S1 Fig. mAP and F1 values for training sets of varied sizes. The size of the circles corresponds to the size of the training set.
(DOCX)
