# Supplementary material for: Neural network-based identification for scallops (Pecten maximus) in natural marine habitats
Source: PLoS One. 2025 Jul 28;20(7):e0327824. doi: 10.1371/journal.pone.0327824 (PMC12303352; doi:10.1371/journal.pone.0327824)
Supplement: S1 Appendix — S1 Table. Evaluation of 3 scallop detection models trained with 3 different sets of class labels. S1 Fig. mAP and F1 values for training sets of varied sizes. The size of the circles corresponds to the size of the training set. (DOCX) [file pone.0327824.s001.docx]

**Appendix**

**Table S1. Evaluation of 3 scallop detection models trained with 3 different sets of class labels.**

| Labels | mAP | optimal confidence | precision | recall | F1 |
| --- | --- | --- | --- | --- | --- |
| alive/dead | 0.42 | 0.9 | 0.49 | 0.39 | 0.44 |
| king/queen/dead | 0.41 | 0.81 | 0.44 | 0.44 | 0.44 |
| surface king/buried king/queen/dead | 0.41 | 0.87 | 0.45 | 0.38 | 0.41 |

**Fig S1. mAP and F1 values for training sets of varied sizes.** The size of the circles corresponds to the size of the training set.
